# Supplementary material for: Xist RNA binds select autosomal genes and depends on Repeat B to regulate their expression
Source: bioRxiv. 2024 Oct 17:2024.07.23.604772. Originally published 2024 Jul 23. Preprint. [Version 2] doi: 10.1101/2024.07.23.604772 (PMC11291044; doi:10.1101/2024.07.23.604772)

bioRxiv preprint doi: https://doi.org/10.1101/2024.07.23.604772; this version posted October 17, 2024. The copyright holder for this preprint (which was not certified by peer review) is the author/funder, who has granted bioRxiv a license to display the preprint in perpetuity. It is made available under aCC-BY 4.0 International license.

SUPPLEMENTAL FIGURE LEGENDS

Figure S1. Xist binds autosomal genes in trans.

A. Spearman (rank-based relationship) and Pearson (linear relationship) correlation analysis show that a strong positive correlation between the two biological CHART sequencing.

B. Xist CHART-seq reads percent of autosomes and Chromosome X in WT and ΔRepB female ES cells at day 0, day 4, day 7, and day 14. ES day 7 exhibits the highest Xist coverage. Sense probe and male ES cells are used as a control.

C. Principal Component Analysis (PCA) of CHART-seq reads include Xist, sense, and input in WT and ΔRepB female, and male ES cells at day 4. Samples clustering closer together share similar genome-wide coverage patterns.

Figure S2. Xist binds X chromosome during female ES cell differentiation.

A-D. Representative binding signals of Xist RNA on Xist locus (A), genes subjective to XCI such as *Cdkl5* (B), *Mecp2* (C), and escapee genes (*Kdm6a*) (D) in WT female ES cells at day 4 and day 7. WT male ES cells and sense probe are used as control.

E. Representative consecutive binding signals of Xist on chromosome X (~320 kb) in WT female ES cells at day 4 and day 7. WT male ES cells are used as control.

F. Representative site-specific binding of Xist on autosome locus (*Stau2*) in WT female ES cells at day 4 and day 7, WT male ES cells are used as control.

Figure S3. Xist binds XCI genes during differentiation.

A. Xist CHART signal coverage on X chromosome genes (XCI-active/inactive, and escapee) in WT female ES cells at day 4. Sense and male ES cells are used as control. P-values are determined using the Wilcoxon rank sum test.

B. Average profile of Xist CHART signal on XCI escapee genes (day 0, 4, 7, and 14) shows that Xist accumulated in the upstream of the promoter but depleted in the escapee gene body.

Figure S4. Xist binds select autosomal genes in trans.

A. IDR (Irreproducible Discovery Rate) analysis to assess the reproducibility of the peaks detected across biological replicates (day 4). The results showed a strong correlation between the replicates, with an IDR threshold of 0.05 (red point > 0.05).

B. Xist peak pattern (MACS2 peak calling) on autosomes in WT and ΔRepB female ES cells at day 4, day 7, and day 14.

bioRxiv preprint doi: https://doi.org/10.1101/2024.07.23.604772; this version posted October 17, 2024. The copyright holder for this preprint (which was not certified by peer review) is the author/funder, who has granted bioRxiv a license to display the preprint in perpetuity. It is made available under aCC-BY 4.0 International license.

C. Representative consecutive binding signals of Xist on chromosome X (~320 kb) in WT and ΔRepB female ES cells at day 4 and day 7.

D. Representative site-specific binding of Xist on autosome locus (*Stau2*) in WT and ΔRepB female ES cells at day 4 and day 7.

E. The Venn diagram illustrates the overlap of peak sites identified in WT and ΔRepB female ES cells at day 4, day 7, and day 14.

Figure S5. Xist binds X-linked genes is Xist's RepB dependent.

A. RNA-seq track patterns of Xist in WT, ΔRepB female, and male ES cells at day 0, day 4, day 7, and day 14.

B. Exemplifying CHART-seq and RNA-Seq patterns of an X-linked gene (*Med12*) at day 7. Change in coverage (Δ1 and Δ2) is shown below (Δ1 for ΔRepB♀ -WT♀, and Δ2 for WT♂ -WT♀).

C. Evaluation of gene expression levels for X-linked genes in WT and ΔRepB female, and male ES cells at day 0. P-values are determined using the Wilcoxon rank sum test.

Figure S6. Genes bound by Xist exhibit higher expression levels and lower H3K27me3 and H2AK119ub binding levels.

A-B. The analysis of gene expression within the 10, 20, 50, and 100-kilobase binding regions of Xist is performed in WT female ES cells at day 7 (B), and day 14 (C), respectively. P-values are determined using the Wilcoxon rank sum test.

C. Average profile plots showing H3K27me3 and H2AK119ub coverage over genes within the 10 and 50-kilobase binding regions of Xist in WT female ES cells.

Figure S7. Example of Xist binding on autosomal genes and influence on gene expression.

This figure illustrates CHART-seq and RNA-seq patterns of autosomal genes, including *Srp9, Brf1, Thra, Cand2, and Kmt2c*, which exhibit Xist binding on different days. *Ces1l*, which lacks Xist binding, is used as a control. Change in coverage (Δ1 and Δ2) is shown below (Δ1 for ΔRepB♀ -WT♀, and Δ2 for WT♂ -WT♀).

Figure S8. Genes not bound by Xist exhibit no changes in gene expression or differences in H3K27me3 and H2AK119ub signals.

bioRxiv preprint doi: https://doi.org/10.1101/2024.07.23.604772; this version posted October 17, 2024. The copyright holder for this preprint (which was not certified by peer review) is the author/funder, who has granted bioRxiv a license to display the preprint in perpetuity. It is made available under aCC-BY 4.0 International license.

A-C. The analysis of gene expression within the 10, 20, 50, and 100-kilobase randomly selected regions is performed in WT female ES cells at day 4 (A), day 7 (B), and day 14 (C), respectively. P-values are determined using the Wilcoxon rank sum test.

D. Average profile plots showing H3K27me3 and H2AK119ub coverage over genes within the 10 and 50-kilobase randomly selected regions in WT female ES cells at different time points.

E. Assessing gene expression levels of Xist non-targets on autosomes in WT, ΔRepB female ES cells, and male ES cells at different time points. P-values are determined using the Wilcoxon rank sum test.

Figure S9. Xist overexpression inhibits select autosomal genes and X-linked genes.

RNA-seq track shows the *Med14* (X-linked gene) (A) and *Bcl7b* (an Xist autosomal target gene) (B) expression levels in differentiated ectopic Xist overexpressed ES cell lines (Tg), the control (Ctrl) was doxycycline-treated wildtype cell. Change in coverage (Δ) is shown below (Tg - Ctrl).

Figure S10. Xist has overlapped autosomal binding peaks in differentiated ES and MEF cells.

A. Coverage of CHART-Seq reads (input, Xist, and sense control) on Chromosome X in WT, ΔRepB, and ΔRepE female MEF cells.

B. The Venn diagram illustrates the overlap of peak sites identified in WT female ES cells at day 4, day 7 and MEF cells.

SUPPLEMENTAL TABLE LEGENDS

Table S1-S3. Xist binding peaks on autosomal regions (MACS2 peak calling) information at day4 (S1), day7 (S2), and day14 (S3) in WT female mouse ES cells.

Table S4-S6. Xist target genes on autosomal regions (10kb among the binding peak) information at day4 (S4), day7 (S5), and day14 (S6) in WT female mouse ES cells.

Table S7-S9. Xist binding peaks on autosomal regions (MACS2 peak calling) information on WT (S7), ΔRepB (S8), and ΔRepE (S9) female MEFs.

Table S10. Xist target genes on autosomal regions (10kb among the binding peak) information on WT female MEFs.

Table S11. Statistical information in this manuscript.

Figure S1

bioRxiv preprint doi: https://doi.org/10.1101/2024.07.23.604772; this version posted October 17, 2024. The copyright holder for this preprint (which was not certified by peer review) is the author/funder, who has granted bioRxiv a license to display the preprint in perpetuity. It is made available under aCC-BY 4.0 International license.

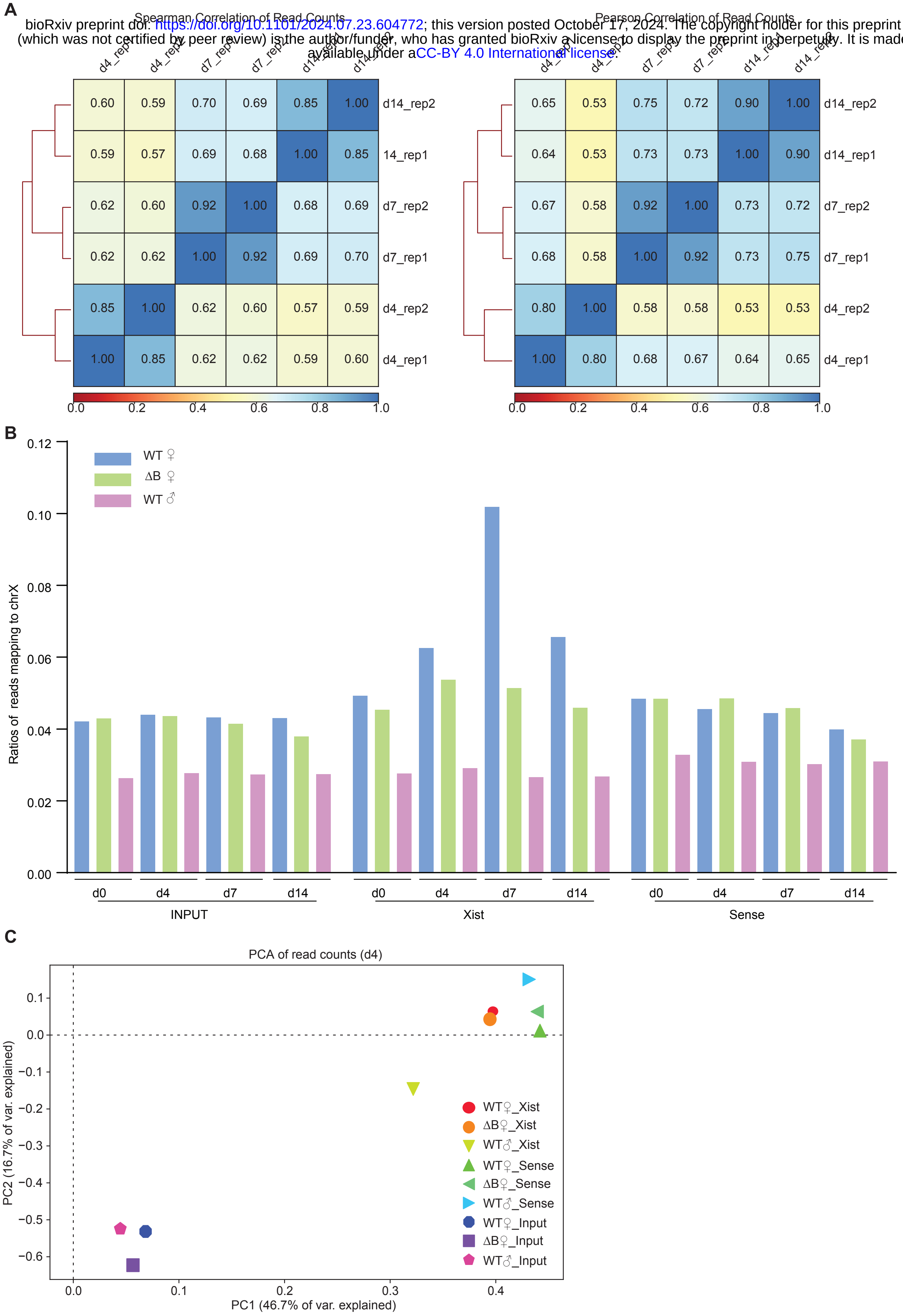

Figure S2

bioRxiv preprint doi: https://doi.org/10.1101/2024.07.23.604772; this version posted October 17, 2024. The copyright holder for this preprint (which was not certified by peer review) is the author/funder, who has granted bioRxiv a license to display the preprint in perpetuity. It is made available under aCC-BY 4.0 International license.

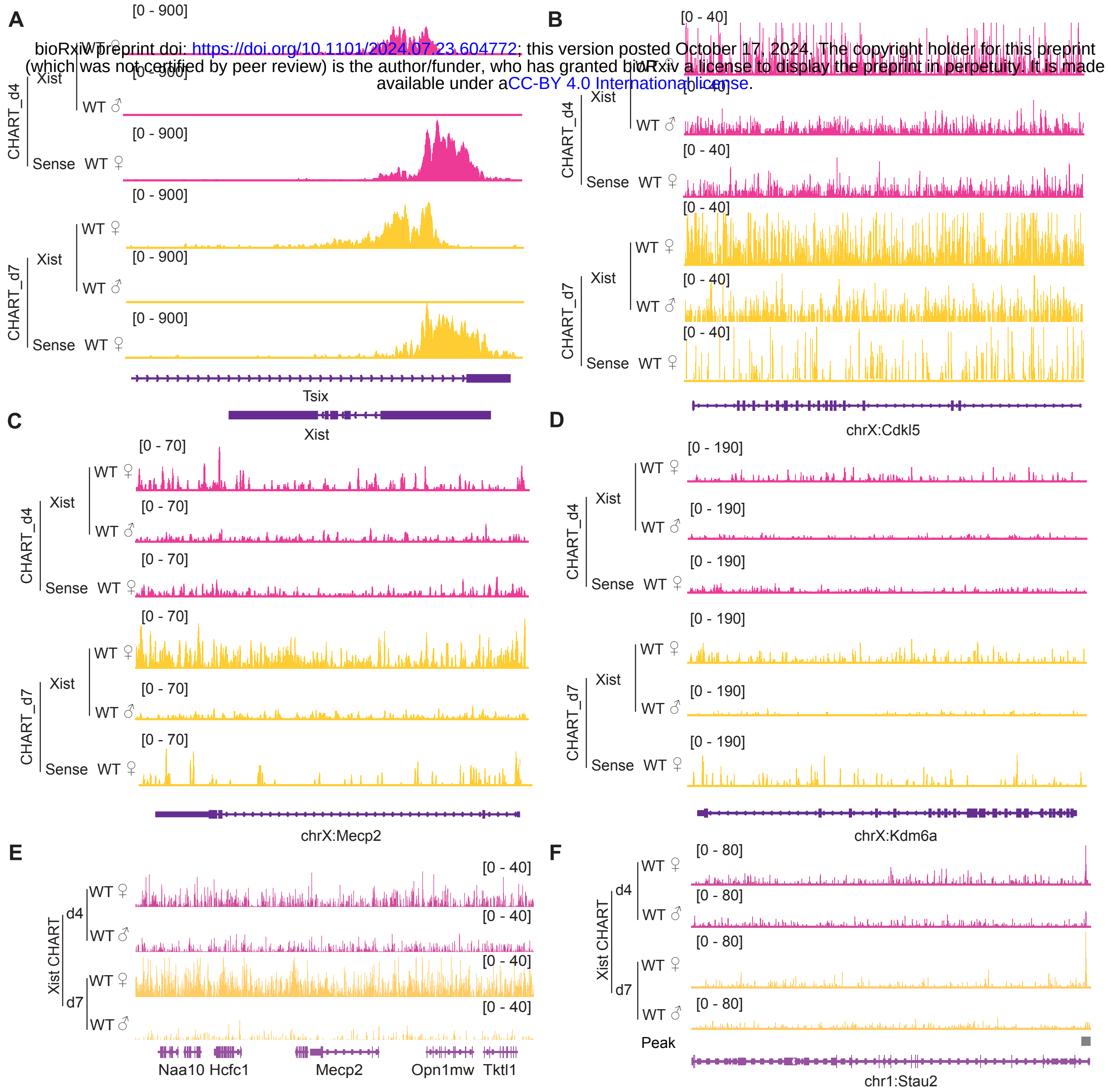

Figure S3

bioRxiv preprint doi: https://doi.org/10.1101/2024.07.23.604772; this version posted October 17, 2024. The copyright holder for this preprint (which was not certified by peer review) is the author/funder, who has granted bioRxiv a license to display the preprint in perpetuity. It is made available under aCC-BY 4.0 International license.

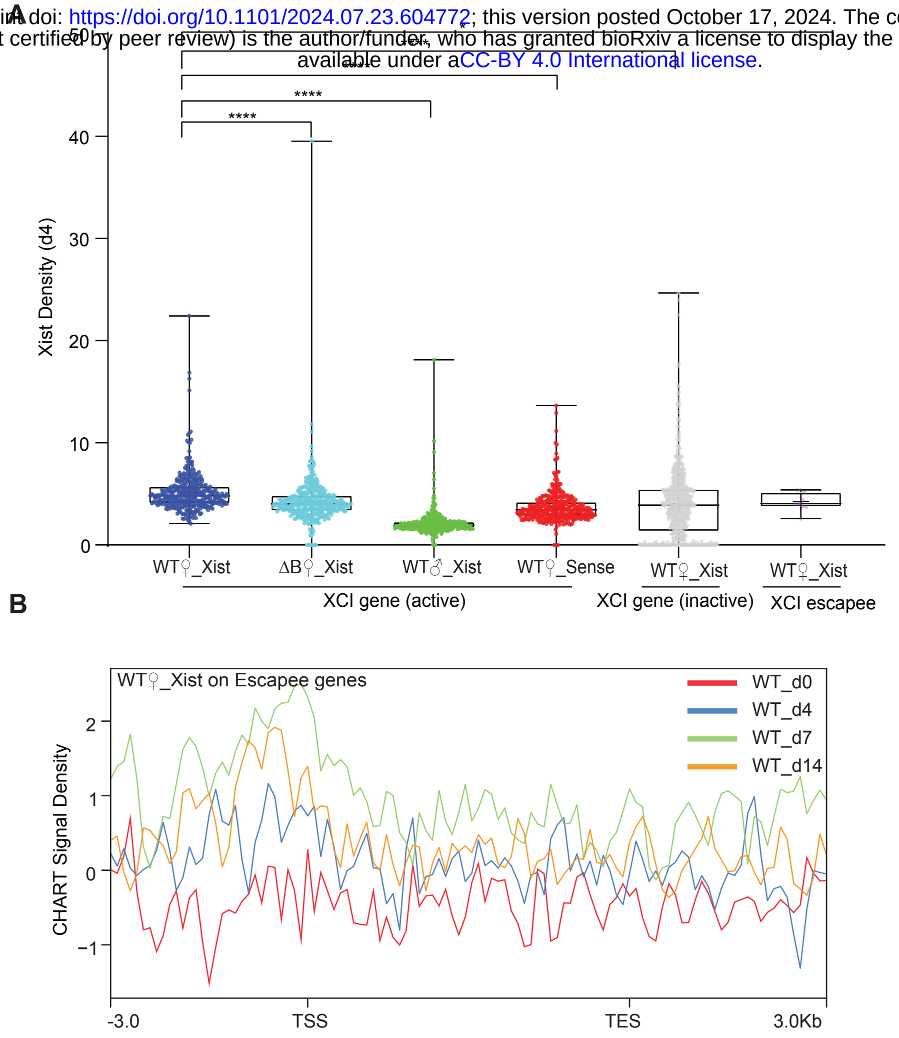

Figure S4

bioRxiv preprint doi: https://doi.org/10.1101/2024.07.23.604772; this version posted October 17, 2024. The copyright holder for this preprint (which was not certified by peer review) is the author/funder, who has granted bioRxiv a license to display the preprint in perpetuity. It is made available under aCC-BY 4.0 International license.

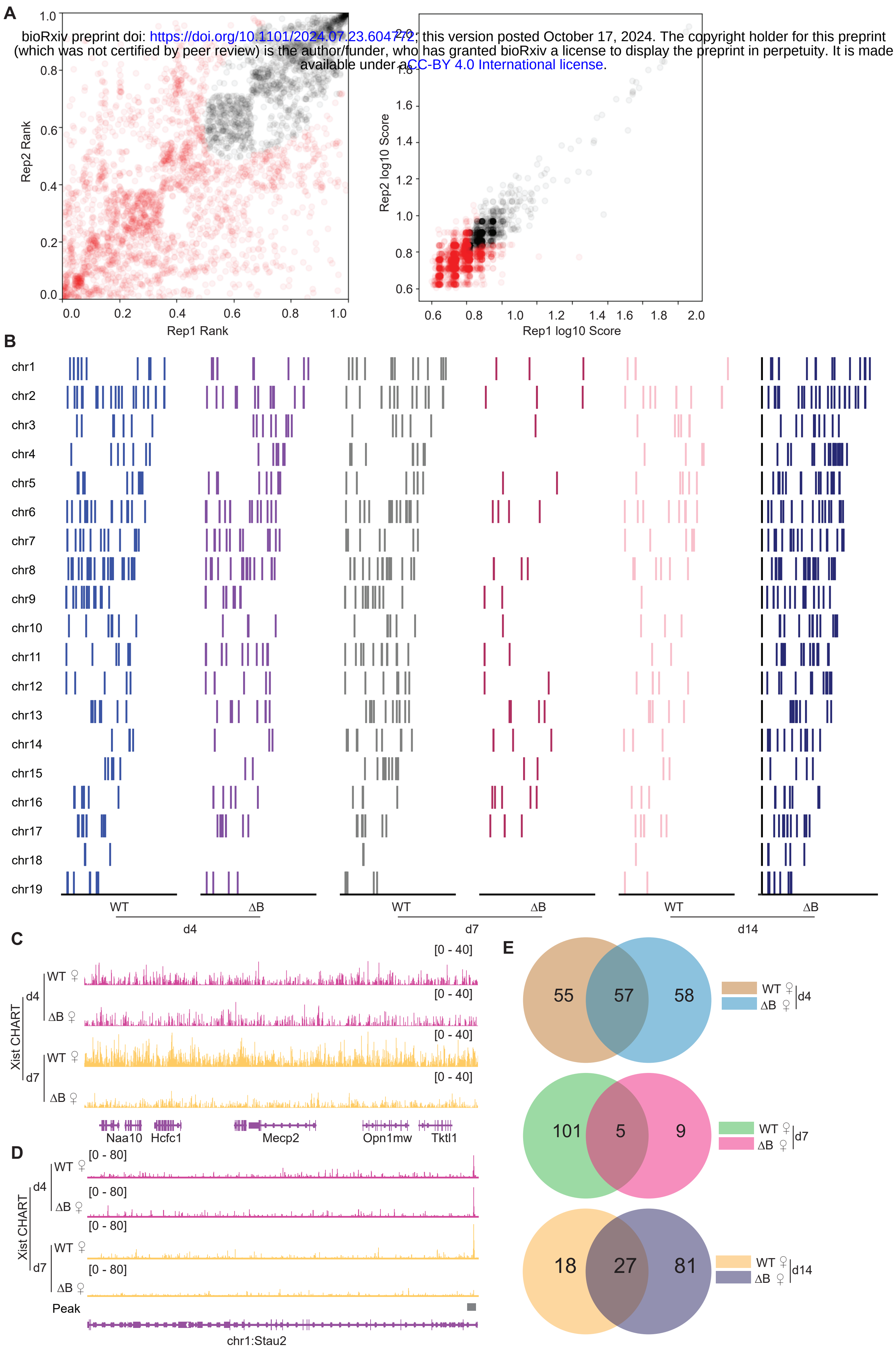

Figure S5

bioRxiv preprint doi: https://doi.org/10.1101/2024.07.23.604772; this version posted October 17, 2024. The copyright holder for this preprint (which was not certified by peer review) is the author/funder, who has granted bioRxiv a license to display the preprint in perpetuity. It is made available under aCC-BY 4.0 International license.

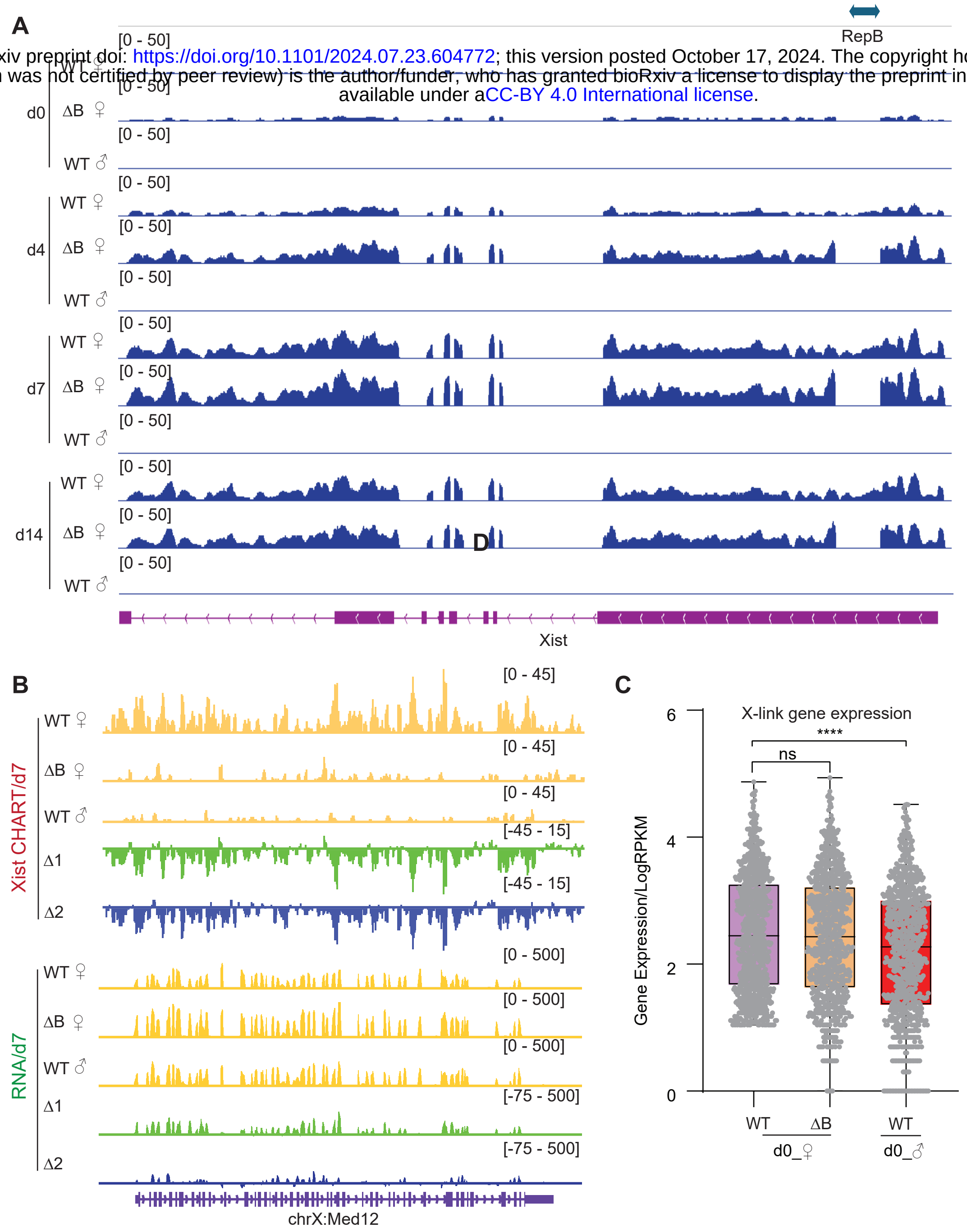

Figure S6

bioRxiv preprint doi: https://doi.org/10.1101/2024.07.23.604772; this version posted October 17, 2024. The copyright holder for this preprint (which was not certified by peer review) is the author/funder, who has granted bioRxiv a license to display the preprint in perpetuity. It is made available under aCC-BY 4.0 International license.

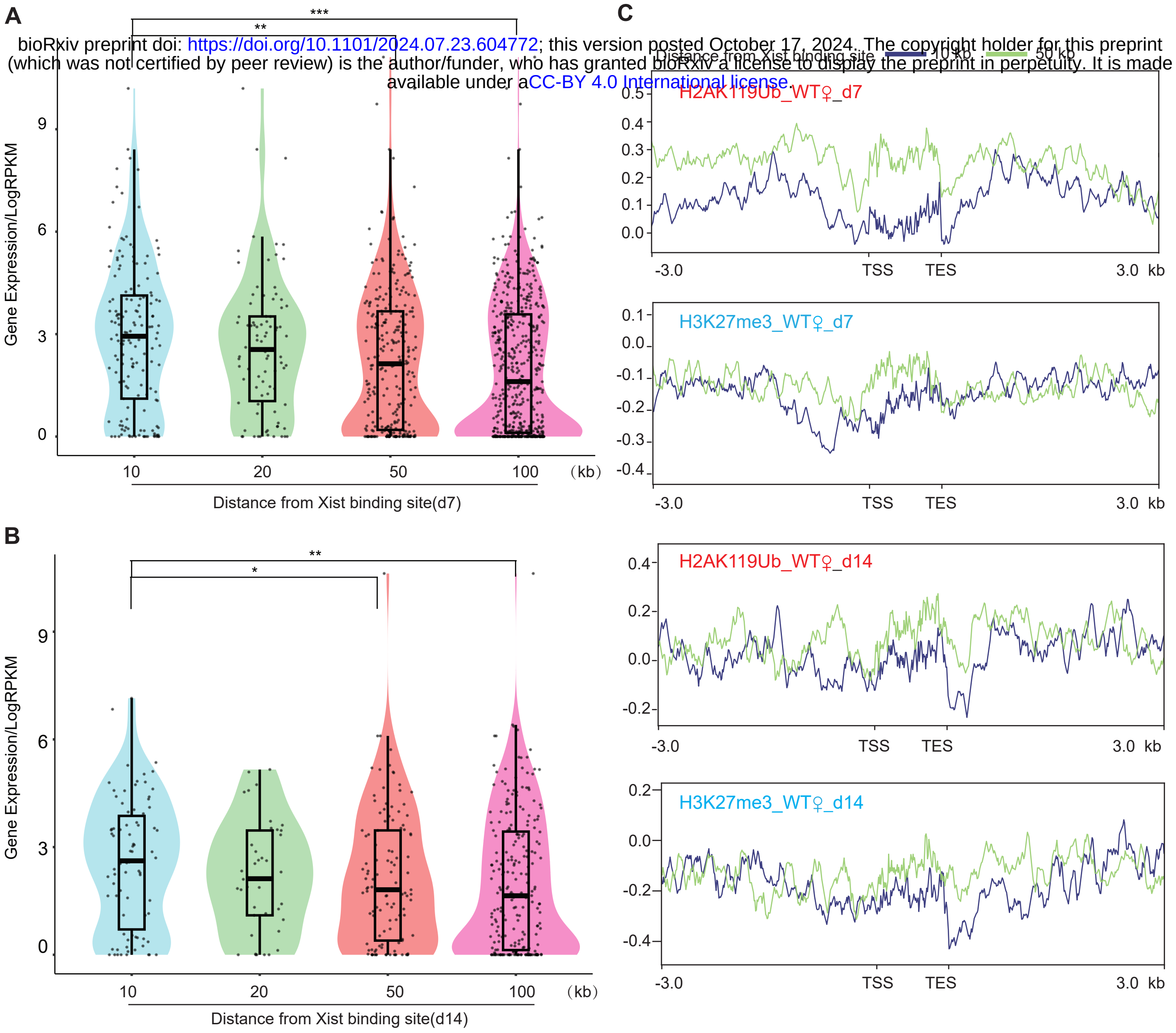

Figure S7

bioRxiv preprint doi: https://doi.org/10.1101/2024.07.23.604772; this version posted October 17, 2024. The copyright holder for this preprint (which was not certified by peer review) is the author/funder, who has granted bioRxiv a license to display the preprint in perpetuity. It is made available under aCC-BY 4.0 International license.

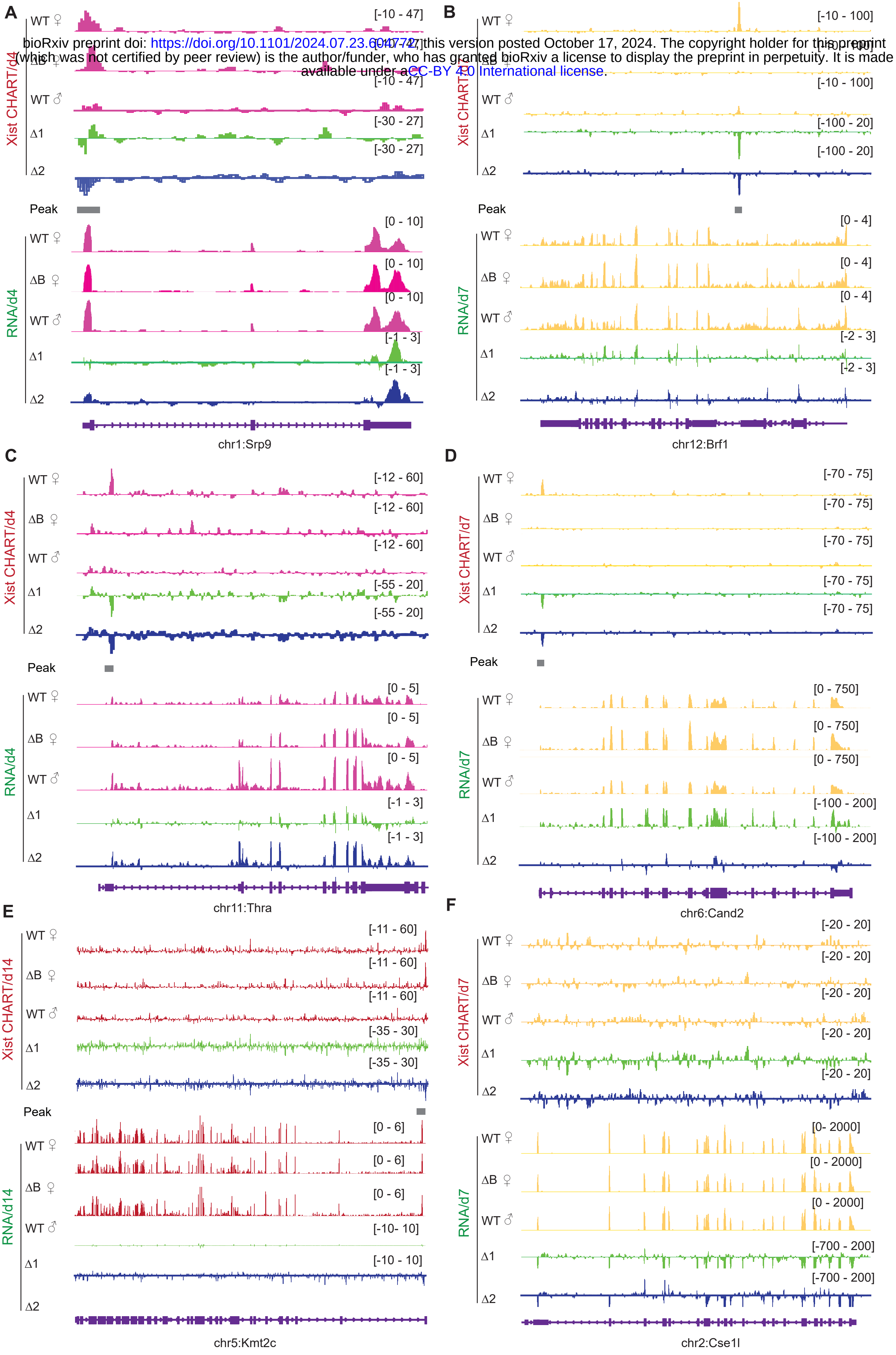

Figure S8

bioRxiv preprint doi: https://doi.org/10.1101/2024.07.23.604772; this version posted October 17, 2024. The copyright holder for this preprint (which was not certified by peer review) is the author/funder, who has granted bioRxiv a license to display the preprint in perpetuity. It is made available under aCC-BY 4.0 International license.

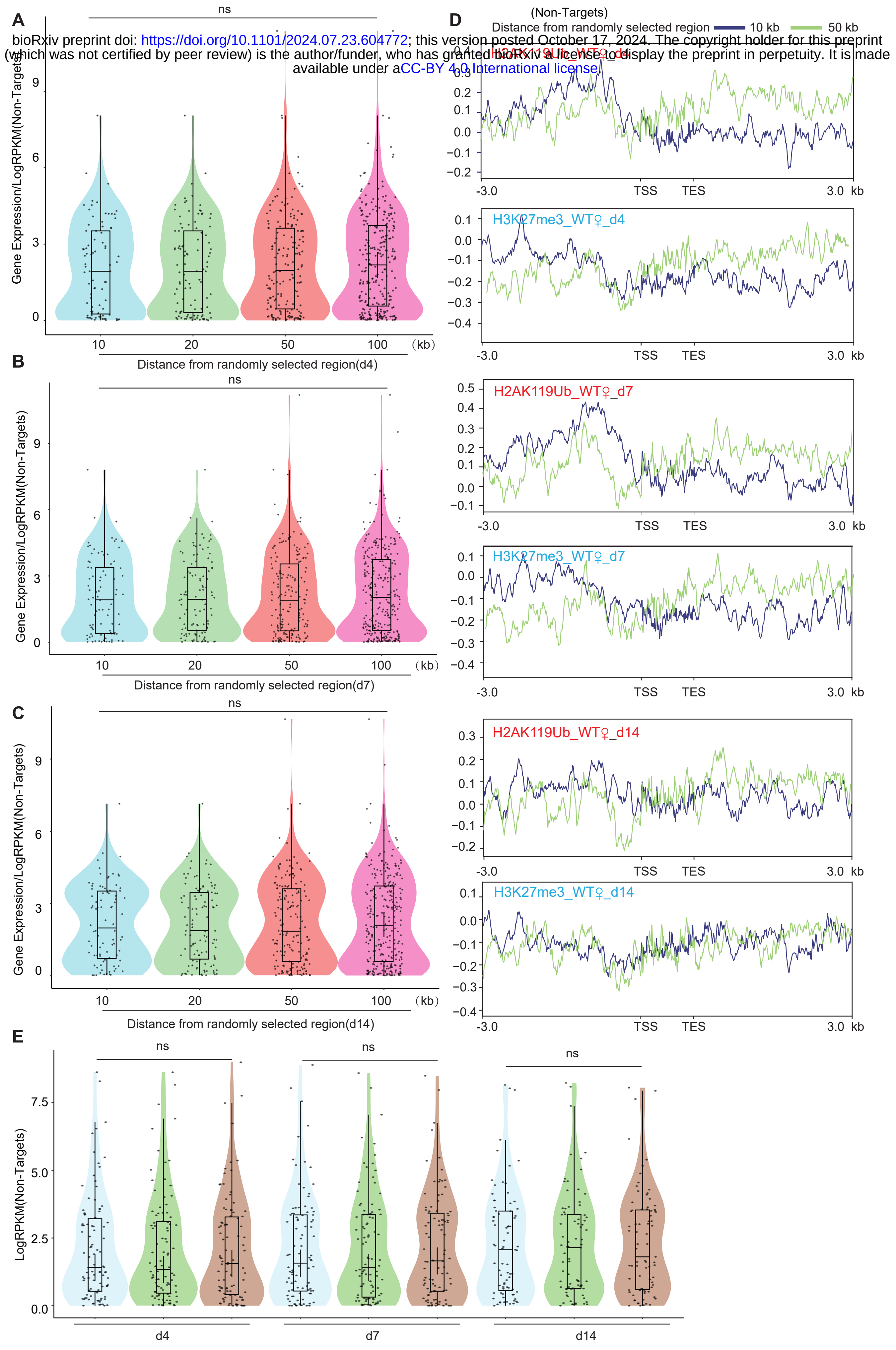

Figure S9

bioRxiv preprint doi: https://doi.org/10.1101/2024.07.23.604772; this version posted October 17, 2024. The copyright holder for this preprint (which was not certified by peer review) is the author/funder, who has granted bioRxiv a license to display the preprint in perpetuity. It is made available under aCC-BY 4.0 International license.

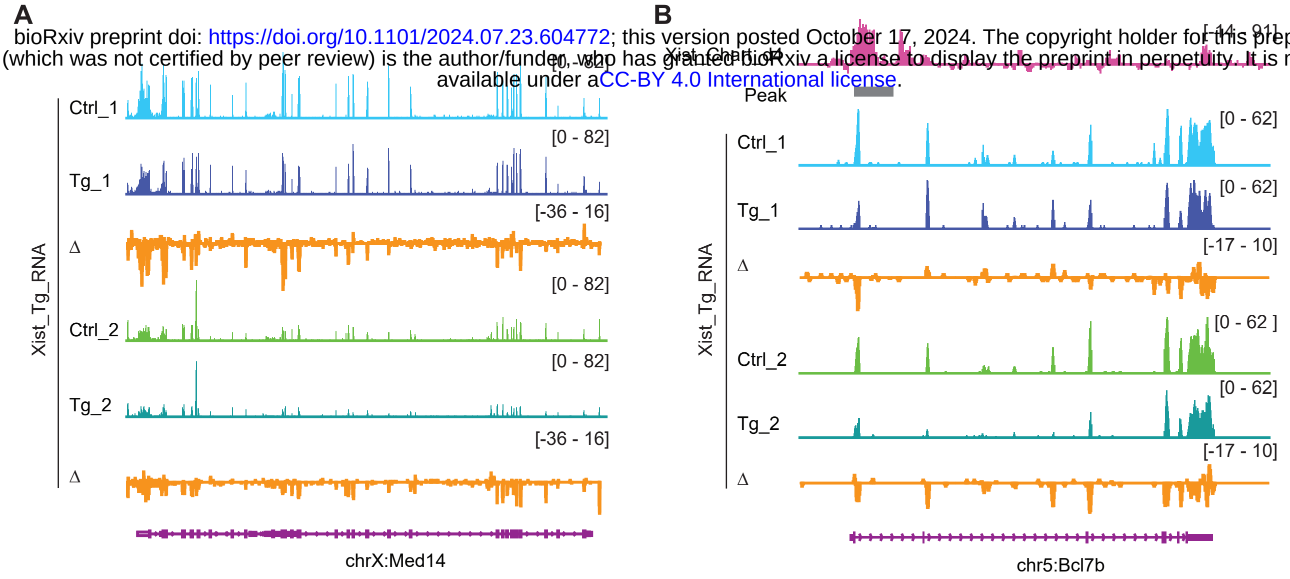

Figure S10

bioRxiv preprint doi: https://doi.org/10.1101/2024.07.23.604772; this version posted October 17, 2024. The copyright holder for this preprint (which was not certified by peer review) is the author/funder, who has granted bioRxiv a license to display the preprint in perpetuity. It is made available under aCC-BY 4.0 International license.

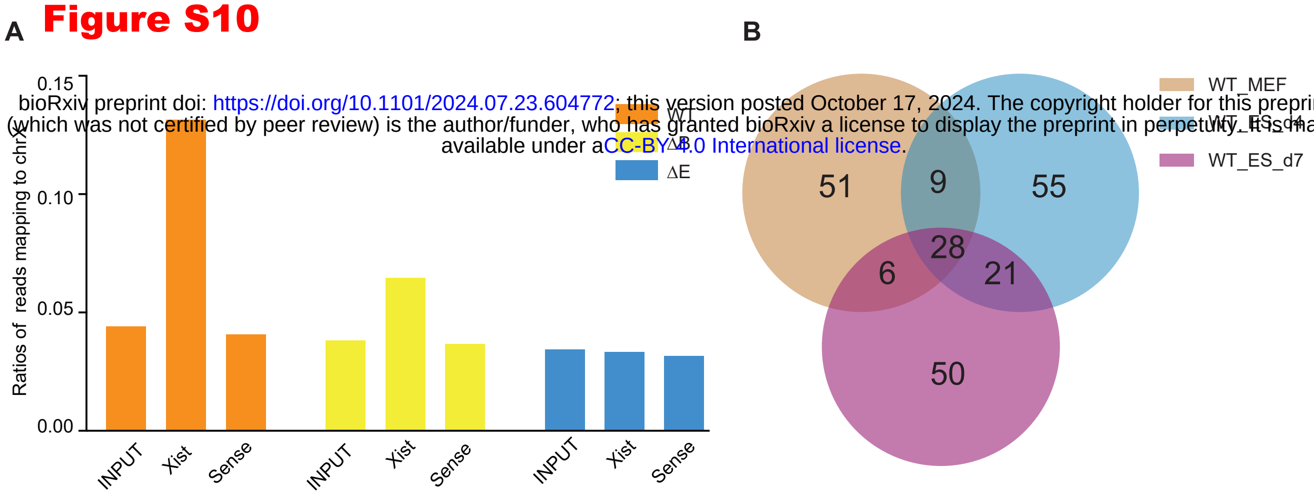

Supplement: Supplement 1 [file NIHPP2024.07.23.604772v2-supplement-1.pdf]
