# Supplementary material for: Xist RNA binds select autosomal genes and depends on Repeat B to regulate their expression
Source: bioRxiv. 2024 Jul 23:2024.07.23.604772. Preprint. [Version 1] doi: 10.1101/2024.07.23.604772 (PMC11291044; doi:10.1101/2024.07.23.604772)

bioRxiv preprint doi: https://doi.org/10.1101/2024.07.23.604772; this version posted July 23, 2024. The copyright holder for this preprint (which was not certified by peer review) is the author/funder, who has granted bioRxiv a license to display the preprint in perpetuity. It is made available under aCC-BY 4.0 International license.

SUPPLEMENTAL FIGURE LEGENDS

Figure S1. Xist binds select autosomal genes in trans.

A. Xist CHART-seq reads percent of autosomes and Chromosome X in WT and ΔRepB female ES cells. ES day 7 exhibits the highest Xist coverage. Sense probe and male ES cells are used as a control. **B-E.** Representative binding signals of Xist RNA on Xist locus (B), genes such to XCI such as *Cdkl5* (C), *Mecp2* (D), and escapee genes (*Kdm6a*) (E) in WT female ES cells on day 4 and day 7. WT male ES cells and sense probe are used as control.

Figure S2. Genes bound by Xist exhibit higher expression levels and lower H3K27me3 and H2AK119ub binding levels.

A. Xist peak pattern (MACS2 peak calling) on autosomes in WT and ΔRepB female ES cells.

B-C. The analysis of gene expression within the 10, 20, 50, and 100-kilobase binding regions of Xist is performed in WT female ES cells at day 7 (B), and day 14 (C), respectively.

D. Profile plots showing H3K27me3 and H2AK119ub coverage over genes within the 10 and 50-kilobase binding regions of Xist in WT female ES cells.

Figure S3. Example of Xist binding on autosomal genes and influence on gene expression.

This figure illustrates CHART-seq and RNA-seq patterns of autosomal genes, including *Srp9, Brf1, Thra, Cand2, and Kmt2c*, which exhibit Xist binding on different days. *Ces1l*, which lacks Xist binding, is used as a control. Change in coverage (Δ1 and Δ2) is shown below (Δ1 for ΔRepB♀ - WT♀, and Δ2 for WT♂ -WT♀).

Figure S4. Genes not bound by Xist exhibit no changes in gene expression or differences in H3K27me3 and H2AK119ub signals.

A-C. The analysis of gene expression within the 10, 20, 50, and 100-kilobase randomly selected regions is performed in WT female ES cells at day 4 (A), day 7 (B), and day 14 (C), respectively.

D. Profile plots showing H3K27me3 and H2AK119ub coverage over genes within the 10 and 50-kilobase randomly selected regions in WT female ES cells at different time points.

bioRxiv preprint doi: https://doi.org/10.1101/2024.07.23.604772; this version posted July 23, 2024. The copyright holder for this preprint (which was not certified by peer review) is the author/funder, who has granted bioRxiv a license to display the preprint in perpetuity. It is made available under aCC-BY 4.0 International license.

E. Assessing gene expression levels of Xist non-targets on autosomes in WT, ΔRepB female ES cells, and male ES cells at different time points.

FigureS1

bioRxiv preprint doi: https://doi.org/10.1101/2024.07.23.604772; this version posted July 23, 2024. The copyright holder for this preprint (which was not certified by peer review) is the author/funder, who has granted bioRxiv a license to display the preprint in perpetuity. It is made available under aCC-BY 4.0 International license.

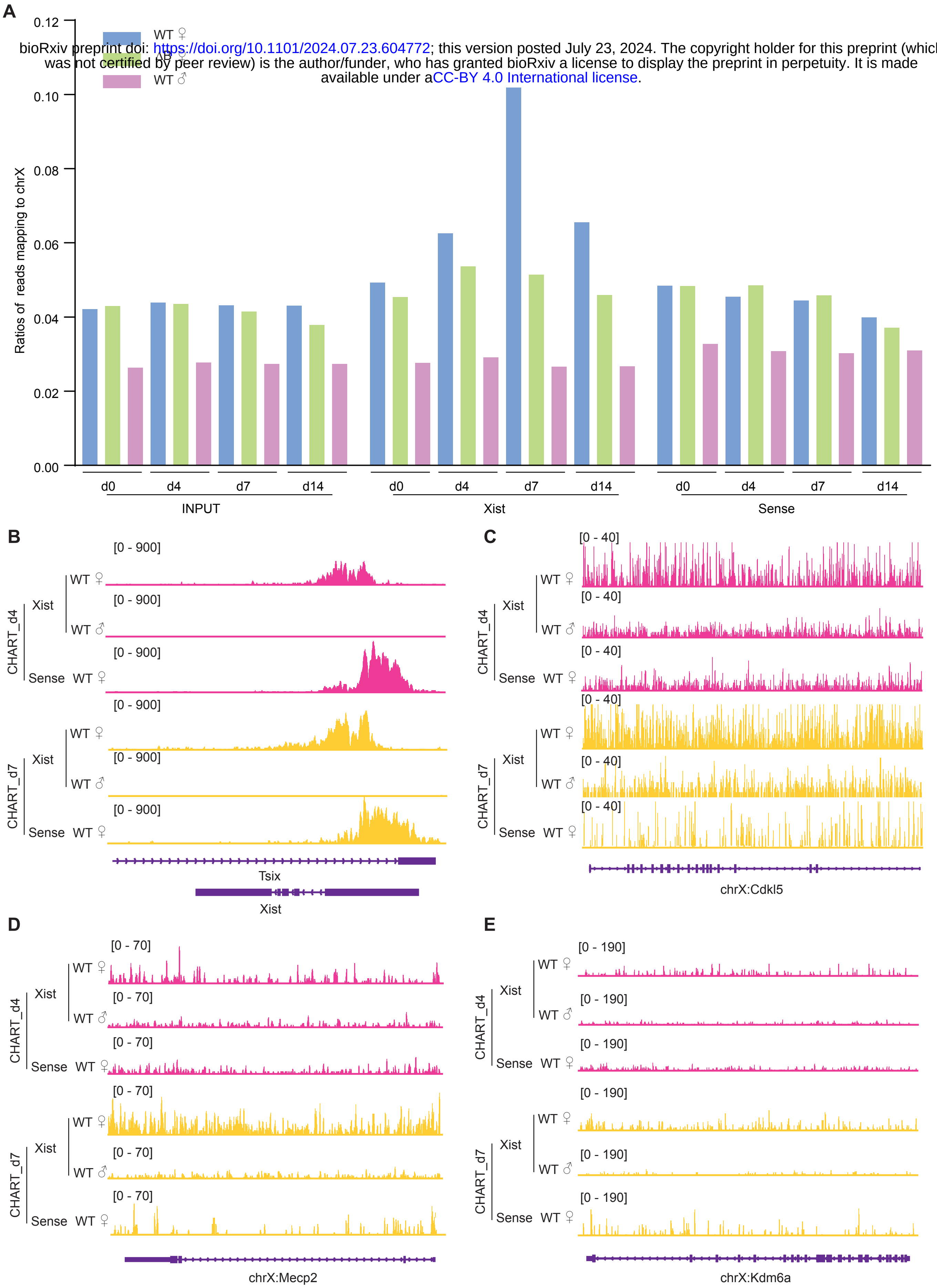

FigureS2

bioRxiv preprint doi: https://doi.org/10.1101/2024.07.23.604772; this version posted July 23, 2024. The copyright holder for this preprint (which was not certified by peer review) is the author/funder, who has granted bioRxiv a license to display the preprint in perpetuity. It is made available under aCC-BY 4.0 International license.

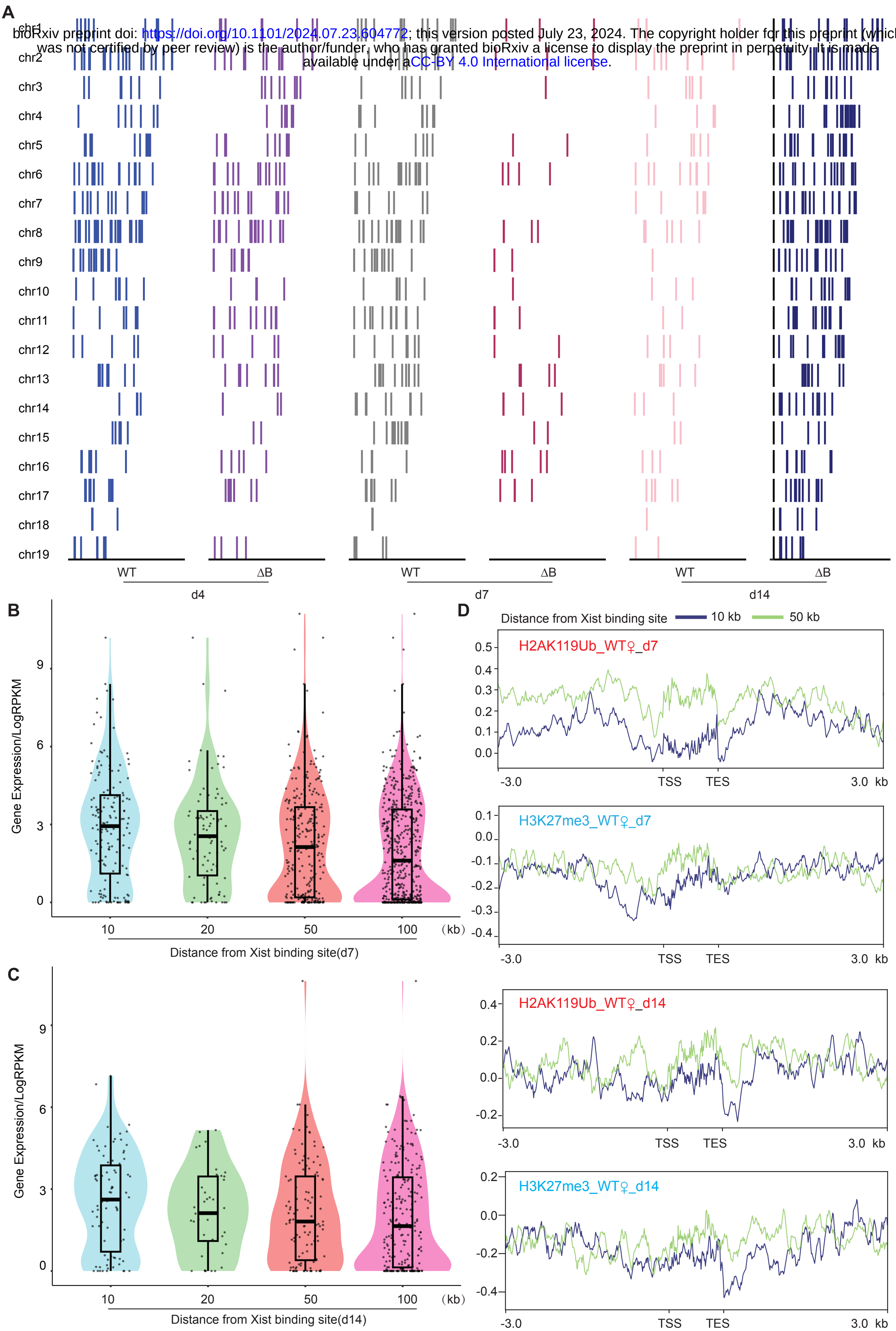

FigureS3

bioRxiv preprint doi: https://doi.org/10.1101/2024.07.23.604772; this version posted July 23, 2024. The copyright holder for this preprint (which was not certified by peer review) is the author/funder, who has granted bioRxiv a license to display the preprint in perpetuity. It is made available under aCC-BY 4.0 International license.

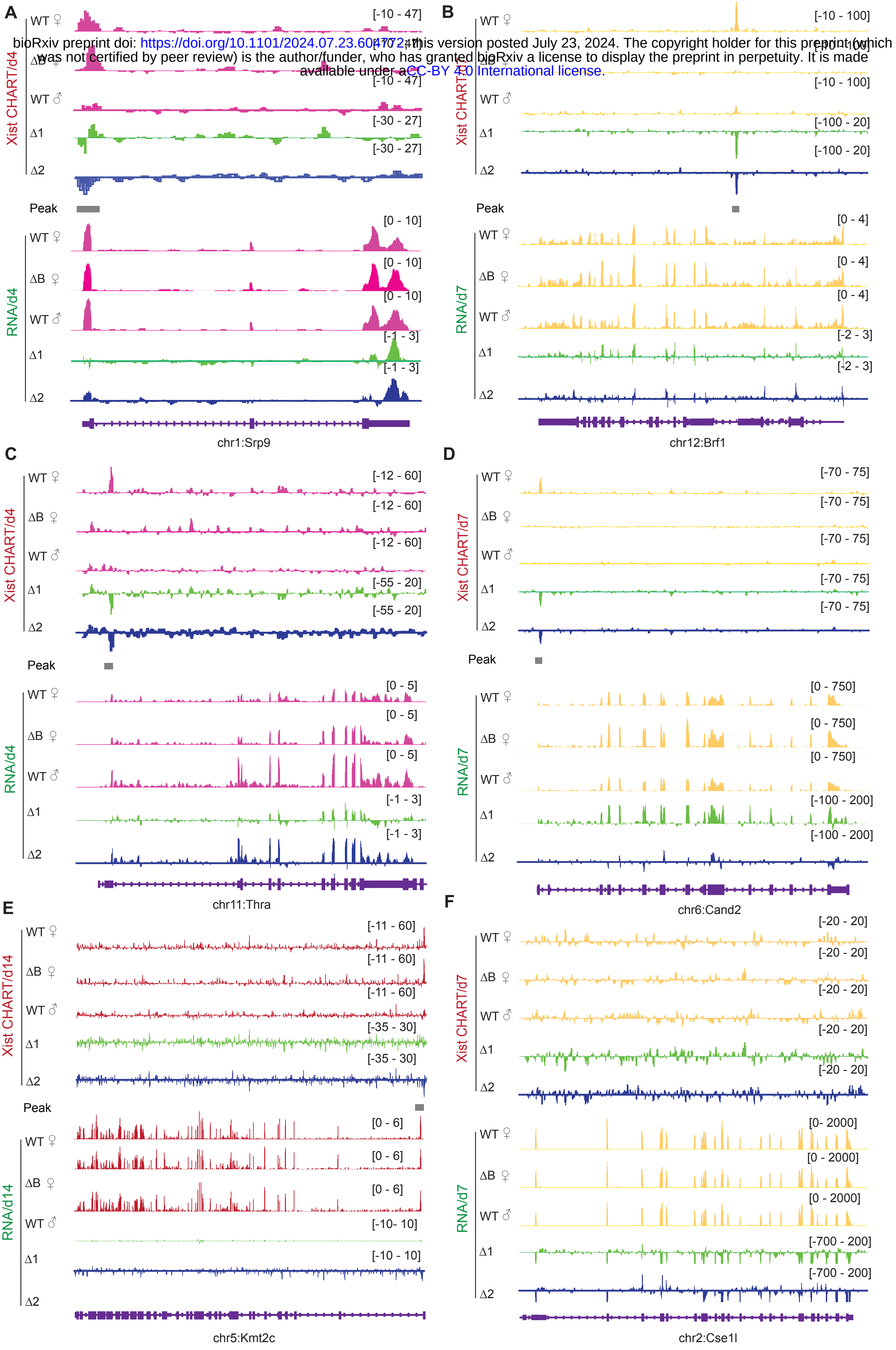

FigureS4

bioRxiv preprint doi: https://doi.org/10.1101/2024.07.23.604772; this version posted July 23, 2024. The copyright holder for this preprint (which was not certified by peer review) is the author/funder, who has granted bioRxiv a license to display the preprint in perpetuity. It is made available under aCC-BY 4.0 International license.

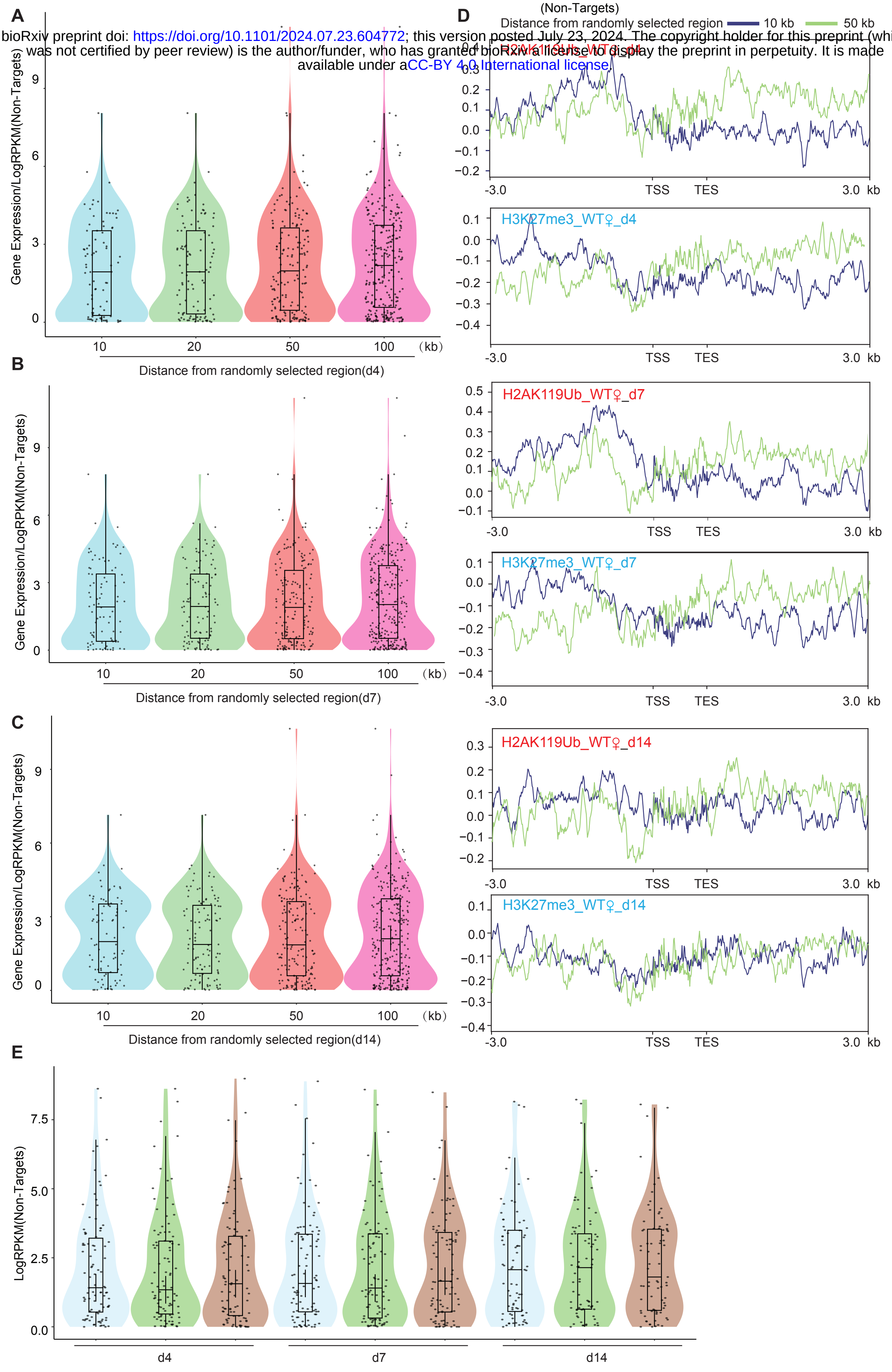

Supplement: Supplementary file 1 [file NIHPP2024.07.23.604772v1-supplement-1.pdf]
